# Targeting Tyrosyl-DNA phosphodiesterase I to enhance toxicity of phosphodiester linked DNA-adducts

**DOI:** 10.20517/cdr.2019.91

**Published:** 2019-12-19

**Authors:** Evan J. Brettrager, Robert C.A.M. van Waardenburg

**Affiliations:** Department of Pharmacology and Toxicology, University of Alabama at Birmingham, Birmingham, AL 35294-0019, USA.

**Keywords:** Tdp1, small molecules, DNA topoisomerases, Camptothecins, oxidative DNA damage, DNA adducts, Etoposide, chain terminating nucleotides/nucleoside analogs, DNA metabolism, drug development

## Abstract

Our genomic DNA is under constant assault from endogenous and exogenous sources, which needs to be resolved to maintain cellular homeostasis. The eukaryotic DNA repair enzyme Tyrosyl-DNA phosphodiesterase I (Tdp1) catalyzes the hydrolysis of phosphodiester bonds that covalently link adducts to DNA-ends. Tdp1 utilizes two catalytic histidines to resolve a growing list of DNA-adducts. These DNA-adducts can be divided into two groups: small adducts, including oxidized nucleotides, RNA, and non-canonical nucleoside analogs, and large adducts, such as (drug-stabilized) topoisomerase- DNA covalent complexes or failed Schiff base reactions as occur between PARP1 and DNA. Many Tdp1 substrates are generated by chemotherapeutics linking Tdp1 to cancer drug resistance, making a compelling argument to develop small molecules that target Tdp1 as potential novel therapeutic agents. Tdp1’s unique catalytic cycle, which is centered on the formation of Tdp1-DNA covalent reaction intermediate, allows for two principally different targeting strategies: (1) catalytic inhibition of Tdp1 catalysis to prevent Tdp1-mediated repair of DNA-adducts that enhances the effectivity of chemotherapeutics; and (2) poisoning of Tdp1 by stabilization of the Tdp1- DNA covalent reaction intermediate, which would increase the half-life of a potentially toxic DNA-adduct by preventing its resolution, analogous to topoisomerase targeted poisons such as topotecan or etoposide. The catalytic Tdp1 mutant that forms the molecular basis of the autosomal recessive neurodegenerative disease spinocerebellar ataxia with axonal neuropathy best illustrates this concept; however, no small molecules have been reported for this strategy. Herein, we concisely discuss the development of Tdp1 catalytic inhibitors and their results.

## Introduction

Tyrosyl-DNA phosphodiesterase I (Tdp1) is a eukaryotic DNA repair enzyme which is a member of the phospholipase D superfamily and hydrolyzes the phosphodiester bond that links an adduct to the end of a nicked DNA strand^[1-3]^. Tdp1 was discovered as an enzyme activity able to hydrolyze a 3’ phospho-tyrosyl linkage, which is the chemical bond between the active site tyrosine of Tyrosine-recombinases and eukaryotic DNA Topoisomerase I (Topo1), and the 3’ phosphoryl-end of a DNA strand^[3]^. Over the last two decades, a broad spectrum of phosphodiester linked 3’ and 5’ DNA-adducts were identified as Tdp1 substrates^[4]^. Tdp1 natural substrates can be divided into two groups: small adducts consisting of damaged nucleotides, DNA inserted ribonucleotides, and non-canonical nucleotide/nucleoside analogs, and large adducts including covalent protein-DNA adducts that are generated as a transient reaction intermediate by, for example, DNA topoisomerases and Tdp1, or protein fragments (peptides) as a result of failed Schiff base linked proteins such as proteolytically processed poly (ADP-ribose) polymerase 1 (PARP1)-DNA adducts [Table 1]. These transient protein-DNA adducts can be stabilized by chemotherapeutics including camptothecins (CPT), epipodophyllotoxins (e.g., etoposide), and local DNA perturbations introduced by, for example, irradiation and endogenously generated reactive oxygen species [Table 1]. Moreover, Tdp1 is localized in both the nuclear and mitochondrial compartments to catalyze the hydrolysis of phosphodiester linked DNA adducts^[4-7]^. This reveals the general role of Tdp1 in maintaining nuclear and mitochondrial genome stability and chemotherapeutic-resistance.

**Table 1:** Therapeutic and endogenous agents generating Tdp1 substrates

Agent	Substrate(s)	Ref.
Reactive oxygen species	3’ abasic site	[8-14]
Chain terminating nucleoside analogs^1^	3’ nucleoside adducts	[10]
Irradiation/Bleomycin	3’ phospho-glycolate, 3’ abasic sites	[15-19]
MMS, TMZ^2^	Methylated bases	[9,20]
PARP1 inhibitors^3^	PARP1-DNA Schiff base	[21-23]
Camptothecins^4^	3’ phospho-tyrosine	[2,3]
Etoposide, doxorubicin^5^	5’ phospho-tyrosine	[20,24,25]
Tdp1 mutants	3’ phospho-histidine	[1,16,26-29]

^1^Arcyclovir (ACV), cytarabine (Ara-C), zidovudine (AZT), zalcitabine (ddC), and sapacitabine; ^2^methyl methanesulfonate (MMS) and temozolomide (TMZ); ^3^BMN673, Olaparib, and Rucaparip; ^4^U.S. Food and Drug Administration (FDA) approved analogs: topotecan and irinotecan; ^5^epipodophyllotoxins such as etoposide and anthracyclins, with Doxorubicin as an example, are Topo2-DNA stabilizing agents with different mechanisms of action^[30]^. Tdp1: Tyrosyl-DNA phosphodiesterase I

**Figure 1:**
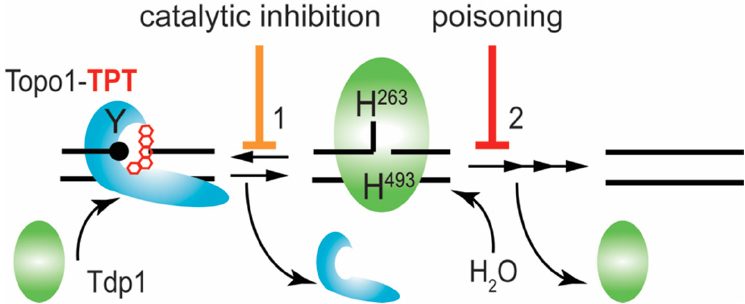
Tdp1 catalytic cycle. Tdp1 utilizes two catalytic histidines to hydrolyze the phosphodiester bond that link adducts to the DNA. Here, we show the example of removal of a Topo1-DNA adduct-3’ phospho-tyrosyl linkage - stabilized by Topotecan. Tdp1 interacts with the Topo1-DNA adduct to initiate Step 1: the nucleophilic attack by His263 - that is, the nucleophilic histidine (in yeast Tdp1 His182) - on the 3’ phospho-tyrosyl linkage forms a 3’ phospho-hystidyl linkage or Tdp1-DNA adduct that releases the tyrosine and by extension Topo1. For Step 2, the general acid/base histidine His493 (His432 in yeast Tdp1) will activate a water molecule to hydrolyze the 3’ phospho-histidyl linkage dissociating Tdp1 from the DNA. However, a single strand nick is left behind by Tdp1 with 5’ hydroxyl and 3’ phosphoryl chemical groups that are processed (reversed) by polynucleotide kinase/phosphatase to facilitate DNA ligase III to regulate the DNA strands. Topo1: topoisomerase I; TPT: topotecan; Tdp1: tyrosyl-DNA phosphodiesterase I

Tdp1 utilizes two highly conserved histidine-lysine-aspartate (HxKx_4_D; x being any amino acid) motifs in two coordinated S_N_2 nucleophilic attacks to hydrolyze the phosphodiester linkage. First, His263 nucleophilically attacks the phosphodiester bond to release the adduct/protein by forming a transient Tdp1-DNA adduct, which is broken by the general acid/base His493 mediated hydrolysis via activation of water releasing Tdp1 from the DNA-end [Figure 1]^[2,3,11,16,26,28,31,32]^. The intriguing fact is that cells risk the formation of second potentially toxic enzyme-DNA reaction intermediate to resolve the primary toxic insult. This potential danger to the cell is highlighted by the catalytic human Tdp1 (H493R) mutant, which is the molecular basis for the rare autosomal recessive neurodegenerative disease spinocerebellar ataxia with axonal neuropathy (SCAN1)^[29,33]^. Tdp1 is expressed in all human tissues at a low level, probably due to its potential danger. However, elevated levels of Tdp1 are detected in a heterologous distribution in virtually all tumors^[7,34]^. Elevated Tdp1 levels stimulate chromosome instability^[35]^ and increase cell sensitivity to DNA damaging agents^[24,26,35-37]^. Thought-provoking is that deletion of Tdp1 in yeast, DT40 chicken cells, HEK293 cells, and mice also results in enhanced cell sensitivity to DNA damaging agents^[2,5,16,20,27,31,38-40]^. Overall, this supports the two different therapeutic strategies hypothesized: (1) no Tdp1 activity or catalytic inhibition of Tdp1 that prevents repair of DNA-adducts leads to cytotoxicity; and (2) accumulation of Tdp1-DNA adducts via poisoning/stabilization of the protein-DNA complex, or increase of Tdp1 levels, results in cytotoxicity [Figure 1]. This review focuses on the current development of Tdp1 as a therapeutic target to improve treatment response with FDA approved chemotherapeutics - a topoisomerase targeting drug^[30]^.

## Tdp1 as therapeutic target

The potential of Tdp1 as a therapeutic target for catalytic inhibitors was directly proposed by Nash and co-workers in its discovery paper^[3]^. Over the last five years, the search for Tdp1 inhibitors has been rapidly growing with the therapeutic focus to combine catalytic Tdp1 inhibiting agents with the current FDA-approved Topo1 targeting chemotherapeutics. Besides catalytic inhibition, we champion a second strategy to poison Tdp1 or to pharmacologically stabilize the enzyme-DNA adduct turning Tdp1 into a cellular toxin, similar to topoisomerase inhibitors^[4,30]^. The principle of this strategy is supported by the SCAN1 His^gab^Arg-mutant and other Tdp1 catalytic mutants tested^[1,16,26,28,29,36,37,41]^. Currently, no small molecules for this strategy have been reported and, herein, we focus only on catalytic Tdp1 inhibitors.

## Catalytic inhibitors

To date, many catalytic Tdp1 inhibitors have been identified, yet only a few were tested in cell or cancer models. Interestingly, many of these Tdp1 catalytic inhibitors are based on natural products isolated from fungi, plants, nucleoside analogs, and bile acids showing inhibition in the low micromolar to the high nanomolar range. The first reported catalytic Tdp1 inhibitors were the non-specific transition metals vanadate (VO_4_^3-^) and tungstate at millimolar concentrations and were used to resolve the crystal structure of the transition state complex of hTdp1 catalytic core domain with VO_4_-DNA-hTopo1 peptide fragments^[42-44]^. Vanadate and tungstate are known general inhibitors for protein phosphotyrosyl phosphatases^[45]^. Yves Pommier and collaborators advanced the search for catalytic inhibitors by exploring existing compounds and developing *in vitro*/alpha-screen based high-throughput screens (HTSs) using oligonucleotides with a 3’ phospho-linked fluorescent adduct to mimic the 3’ phospho-tyrosyl bond. They exploited antibiotic ribosomal inhibitors and identified as the first pharmacological active Tdp1 inhibitors Neomycin, an aminoglycoside analog, and non-aminoglycosides such as puromycin and thiostrepton with IC_50_ ranging 2-30 mmol/L^[46]^. They further identified rolitetracycline and potentially other tetracycline analogs to inhibit Tdp1 catalysis at micromolar levels^[47]^. Huang *et al.*^[48]^ successfully translated this observation by repurposing minocycline in combination with irinotecan to treat high grade ovarian cancer cell lines and an orthotopic xenograft mouse model of human ovarian carcinomatosis. Intriguingly, minocycline decreased Tdp1 expression levels that enhanced irinotecan toxicity in platinum resistant cell lines, while *in vivo* this combination reduced micro-metastases to improve overall survival. Why minocycline specifically reduces Tdp1 protein levels and/or inhibits Tdp1 activity as suggested for another tetracycline analog - rolitetracycline - is currently unknown^[47,48]^. How tetracyclines inhibit eukaryotic protein synthesis is still questionable - the most popular explanation is ribosome inhibition^[49]^, but the reduction of one protein (Tdp1) by minocycline remains an enigma.

The diamidine analogue furamidine [2,5-bis (4-amidinophenyl) furan] was the first inhibitor identified in a HTS at National Institutes of Health (NIH)^[50]^. Furamidine is used to treat Trypanosomiasis and leishmaniasis, which express Tdp1, but it is unclear if Tdp1 inhibition is part of the antiparasitic activity^[51]^. An interesting twist is that furamidine combined with irinotecan suppresses murine lupus nephritis^[52]^. Additional HTSs at NIH identified additional micromolar to high nanomolar Tdp1 inhibitors: progesterone derivative NSC88915 (3,20-dioxopregn-4-en-21-yl 4-bromobenzenesulfonate) and phospho-tyrosyl mimetics such as suramin and dephostatin derivative methyl-3,4-dephostatin (3,4-Dihydroxy-*N*-methyl-*N*-nitrosoaniline)^[53,54]^. The Pommier and Wang collaboration identified arylidene thioazolidinone derivatives as high nanomolar inhibitors of Tdp1 catalysis^[55]^.

**Figure 2:**
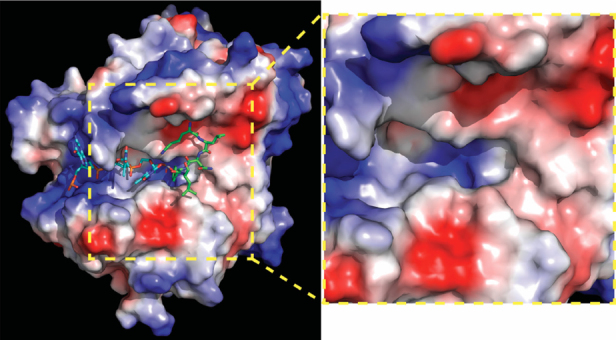
Human Tdp1 electrostatic surface distribution. Electrostatic surface potential of human Tdp1 is shown in a gradient from negative (red) through neutral (white) to positive (blue) and was degenerated by PyMol. Shown in cyan is the DNA and in green the Topo1-peptide fragment that is bonded to the DNA via phosphate. In the used structure, the phosphate was replaced by a vanadate to capture the Tdp1-Topo1-DNA complex (PDB file: 1NOP Davies 2003). A positively charged “DNA-gorge/cleft” fits single strand DNA with the adducted end located in the catalytic pocket from which a “funnel cone” shape pocket emerges that facilitates docking of the protein/peptide adduct. The yellow zoom box highlights the electrostatic charge distribution of the DNA-gorge, catalytic pocket, and funnel cone in which a potential inhibitor will need to bind to prevent Tdp1 interaction with and hydrolyzes of a DNA-adduct. The figure was generated using MacPyMol (DeLano Scientific, San Carlos CA). Topo1: topoisomerase I; Tdp1: tyrosyl-DNA phosphodiesterase I

The identification of non-hydrolysable phospho-tyrosyl mimetics that perfectly docked in the catalytic pocket led to the first in silico-docking screen to classify 46 additional potential Tdp1 inhibitors that dock the catalytic cavities [Figure 2], as such competing with Tdp1 substrates^[56]^. However, these compounds were not verified for their potential biochemical and biological activity. Subsequent in silico-docking screens by other groups did verify their compounds; Gushchina *et al.*^[57]^ found thioether sulfo-heterocyclic linked compounds that docket into the catalytic pocket and inhibited catalysis in the high micromolar range. Waugh’s group recently reported an impressive crystallographic verification of 11 phthalic acids and quinolone-based fragment ligands identified in their in silico-docking screen^[58]^. They demonstrated in silico docking within Tdp1 catalytic pocket, inhibition of Tdp1 catalysis in the mid micromolar to high millimolar range, and resolved 11 independent crystal structures of the Tdp1 catalytic domain in complex with these compounds to confirm their in silico-docking screen results, concluding they identified competitive Tdp1 inhibitors^[58]^. This approach provides a great basis for structure-based drug design to further develop these inhibitors and test them in cell-based/mice models.

However, except for furamidine and minocycline, none of these compounds were verified in cell-based or animal models of cancer. The first report using cell-based screens for Tdp1 inhibitors exploited Tdp1-deficient DT40 chicken cells complemented with and without human TDP1 to identify compounds that demonstrate a synergistic effect with CPT^[22]^. This approach, however, identified PARP1 inhibitors and not Tdp1 inhibitors. The authors identified five compounds after a primary (DT40*tdp1*^-/-^ + hTDP1) and secondary (DT40*tdp1*^-/-^ *vs.* DT40*tdp1*^-/-^ + hTDP1) screen that did not show Tdp1 inhibition but inhibited PARP1-activity analyzed by ELISA and immunoblotting for PARylation. Moreover, this revealed how tricky cell-based screens and the tight cellular interplay of DNA repair-DNA damage response proteins are. This tight interplay can also foster alternative treatment strategies. For example, Pfeifer and collaborators reasoned that Tdp1 inhibitors will be synergistic with CPT but also with PARP1 inhibitors^[34]^. This is mechanistically supported by the reported observation that Tdp1 and PARP1 are epistatic for the repair of Topo1-DNA adducts^[9,59]^. They identified an alkylidene barbiturate derivative [CD00509; 5-(2-Furylmethylidene)-2-thioxohexahydropyrimidine-4,6-dione] in a biochemical-screen and verified this compound in TDP1 and *tdp1*^-/-^ MEF-cells, showing that TDP1-MEFs with CD00509 showed a similar CPT sensitivity as *tdp1*^-/-^ MEFs without CD00509. Moreover, CD00509 combined with CPT or Rucaparib in MCF7 breast cancer cells resulted in more toxicity for both combinations compared to the agent alone^[34]^. Thus, Tdp1 inhibition together with PARP1 inhibition is a successful treatment strategy taking advantage of additional impeding mutations that cancer cells maintain. Current knowledge adds an additional explanation: PARP1 inhibitors not only inhibit PARP1 catalysis but also stabilize the PARP1-DNA intermediate^[60]^, the reaction intermediate of a Schiff base reaction, which itself induces cytotoxicity. Moreover, the phosphodiester bond linking the damaged nucleotide to the PARP1 peptide is a substrate for Tdp1 hydrolyses^[23]^. Pommier teamed up with the Malhorta group and broadened cell-based screening by testing 15 newly synthesized piperidinyl sulfamide derivatives in the NCI60 cell line panel^[61]^. The advantage of this NCI60 screen is that, in addition to screening for compound induced cytotoxicity, it may reveal potential cancer specificity/cell type and may reveal potential response pathways, since the NCI60 panel has been molecular characterized over the years. Moreover, the inclusion of *R*- and *S*-stereoisomers will reveal differences in biological activity, which was observed for the only compound that induced a significant cell toxicity and inhibited Tdp1 catalysis, namely piperidyl sulfamide-18 [NSC750706; (*R*)-Methyl 2-(*N*-(1-(4-fluorobenzyl)piperidin-4-yl)-*N*-(3-fluorophenyl) sulfamoylamino)-3-methyl butanoate], while its S-stereoisomer, NSC764209, induced no phenotype^[61]^.

Another forerunner in the hunt for Tdp1 catalytic inhibitors is Olga Lavrik and her collaborators. These researchers synthesized their derivatives and evaluated the compounds in: (1) Tdp1 catalytic assay; (2) in silico docking; and (3) cell toxicity/growth inhibition studies. They identified a wide variety of chemical scaffolds that include the Benzopentathiepines derivative 2-(Dibutylamino)-*N*-(8-(trifluoromethyl)benzo[f]-[1,2,3,4,5]pentathiepin-6-yl)acetamide, which inhibits catalysis at high nanomolar concentrations and induces MCF7 cytotoxicity (IC_50_ ~ 28 μmol/L) via DNA fragmentation and apoptosis^[62]^. They combined a 7-hydroxycoumarin with monoterpenoid moieties resulting in 7-(((*1S,5R*)-6,6-Dimethylbicyclo[3.1.1]hept-2-en-2-yl)methoxy)-2,3-dihydrocyclopenta[c]chromen-4(*1H*)-one that exhibits a high nanomolar catalytic inhibition (IC_50_ = 675 ± 7 nmol/L), a MCF7 CC_50_ of 180 nmol/L (in combination with CPT) and increased MCF7 CPT sensitivity, but had no effect on RPMI-8226 human multiple melanoma cells that maintain lower Tdp1 levels than MCF7 cells^[63]^. They concluded that the induced toxicity is Tdp1-dependent; however, this would have been better supported by knockdown of Tdp1 levels, since many other different factors, including PARP1 activity, can contribute to a lack of effect/phenotype. Using the Structure-Activity Relationship of octahydro-*2H*-chromen-4-ol scaffold, the Lavrik group developed a series of 3(*4S*)- and 3(*4R*)-diastereomers derivative with different bulky side-groups that inhibit Tdp1 catalysis in the low micromolar range while in silico-docking showed that each of these six ligands binds Tdp1 in more than one location within the Tdp1 catalytic cavity [Figure 2]^[64]^. Recently, Lavrik and colleagues reported 15 monoterpenoid and adamantane fragments, which are able to inhibit Tdp1 catalysis (0.86-4.08 μmol/L). Of these 15 fragments, 3,7-Dimethyloctyl adamantane-1-carboxylate in combination with topotecan induced synergistic toxicity in A549 human lung carcinoma cells^[65]^.

**Figure 3:**
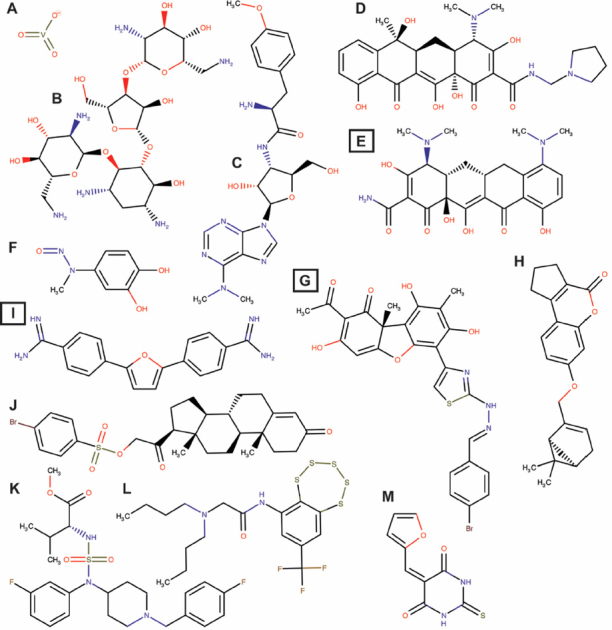
Structures of potential Tdp1 inhibitors. A: Vanadate; B: Neomycin; C: Puromycin; D: Rolitetracycline; E: Minocycline; F: methyl-3,4-dephostatin; G: (R,E)-2-acetyl-6-(2-(2-(4-*bromobenzyliden)hydrazinyl*)*thiazole-4-yl*)-3,7,9-trihydroxy-8,9b-*dimethyl dibenzo*[b,d]furan-1(9bH)-one; H: 7-(((1S,5R)-6,6-Dimethylbicyclo[3.1.1]hept-2-en-2-yl)methoxy)-2,3-*dihydrocyclopenta*[c]chromen-4(1H)-one; J: 3,20-Dioxopregn-4-en-21-yl 4-bromobenzenesulfonate; K: 2-(Dibutylamino)-N-(8-(trifluoromethyl)benzo[f]-[1,2,3,4,5] pentathiepin-6-yl)acetamide; L: (R)-*Methyl 2*-(N-(1-(4-fluorobenzyl)piperidin-4-yl)-N-(3-fluorophenyl) *sulfamoyl amino*)-3-methylbutanoate; M: 5-(2-*Furyl Methylidene*)-2-*thioxo hexahydro pyrimidine*-4,6-dione. Box letters are compounds tested *in vivo*. Chemical structures were drawn using MarvinSketch (17.3.13.0) ChemAxon (http://www.chemaxon.com)

In addition to arrays of synthesized compounds, Lavrik and co-workers also utilized natural product scaffolds in their search for potential Tdp1 catalytic inhibitors. They synthesized 29 aryliden- and hetarylidenfuranone derivatives of usnic acid (a metabolite found in various lichens) that inhibit Tdp1 in the low nanomolar range. These compounds also induced A549 cytotoxicity with IC_50_ between 5 and 20 μmol/L and potentiated topotecan toxicity^[66]^. Their subsequent synthesized hydrazinothiazole usnic acid derivative (*R,E*)-2-acetyl-6-(2-(2-(4-bromobenzyliden)hydrazinyl)thiazol-4-yl)-3,7,9-trihydroxy-8, 9b-dimethyldibenzo[b,d]furan-1(9bH)-one is an effective Tdp1 catalytic inhibitor that increased topotecan toxicity in a Lewis lung carcinoma cell model and was the first potential Tdp1 inhibitor to show, in combination with topotecan, an anti-tumor and anti-metastatic effect in a mouse model of Lewis Lung Carcinoma^[67]^. This compound is now entering the preclinical trial phase. This group also used semi-synthetic derivatives of bile acids and disaccharide nucleosides as a scaffold for the development of Tdp1 catalytic inhibitors^[68,69]^. The bile acid derivatives were tested by in silico-docking and in a catalytic assay showing inhibition in the 300 to 500 nmol/L ranges with *N*-(2’’-(3’,5’-Di-tret-buthyl-4’-hydroxyphenyl)-ethyl)-3a,12a-diacetoxy-5b-cholan-24-amide as the most promising compound^[69]^. Disaccharide nucleosides were explored as Tdp1 inhibitors following reports showing that pyrimidine disaccharide derivatives including nicotinamide adenine dinucleotide (NAD^+^)-mimetics catalytically inhibited PARP1^[70]^ and that PARP1 synthesized free PAR-monomers and -polymers that inhibit, for example, XPC-RAD23B^[71]^. Disaccharide nucleoside analogs inhibited wild type Tdp1 catalysis (low micromolar to high nanomolar range) but interestingly not the Tdp1H493R-SCAN1-mutant^[68]^. Why these compounds do not show inhibition of the SCAN1-mutant enzyme is unknown and cannot be explained from the reported experimental results. However, some of these compounds potentiated topotecan induced toxicity in A549 cells and non-cancerous WI-38 (fibroblasts derived from lung tissue of a three months gestation female fetus) cells, suggesting to induce “normal” cell toxicity. These active derivatives can be divided into three classes: (1) (1’-2’)-glycosidic bond (2’-*O*-pentafuranosyl nucleosides); (2) b(1’-3’)-glycosidic bond (3’-*O*-b-*D*-ribofuranosyl nucleosides); and (3) b(1’-5’)-glycosidic bond (5’-*O*-β-*D*-ribofuranosyl nucleosides). They induce catalytic inhibition in the low micromolar to high nanomolar range, but need further development^[68]^. Quinn and co-workers exploited 3,4-dimethoxyphenol-1-β-*D*-(6’-*O*-galloyl) glucopyranoside and 3-(4-hydroxy-3-methoxyphenyl)propane-1,2-diol2-β-*D*-(6-*O*-galloyl) glucopyranoside from Macropteranthes leichhardtii, and achyrodimer F from the teleomorphic fungus family Cortinariaceae. Both compounds inhibit Tdp1 in the low micromolar range^[72,73]^. Takagi *et al.*^[74]^ isolated JBIR21 from an unidentified anamorphic fungus RF-13305 culture that showed catalytic inhibition with IC_50_ of 18 μmol/L and induced growth inhibition of cervical carcinoma HeLa cells, malignant mesothelioma NCI-H2052 cells, colon adenocarcinoma HT-29 cells, and lymphoblastoid namalva cells with an IC_50_ range of 3.5-3 μmol/L. JBIR21 also showed an antitumor effect in a HT-29 xenograft model (treatment-to-control ratio of 0.51) without noticeable toxicity or other adverse effects, suggesting that JBIR21 forms a highly potential scaffold for further development of a clinically applicable compound. Figure 3 shows examples of structures of potential Tdp1 inhibitors discussed above.

## Conclusion

Over the last two decades, the development of Tdp1 catalytic inhibitors has produced active compounds that showed a high potential to be tested in (pre-)clinical trials. Although these compounds were originally selected for their ability to inhibit Tdp1 catalysis and modeled-docking of the compounds into the Tdp1 catalytic pocket, the more current and promising compounds were tested in combination with DNA Topo1 inhibitors (topotecan or irinotecan) in cell and xenograft mouse models of cancer. However, Tdp1 specificity is still unclear and has not been addressed yet for all these compounds. Moreover, the potential development of Tdp1-poisons, compounds that selectively increase the lifetime of Tdp1-DNA adducts similar to Tdp1 catalytic mutants such as the SCAN1 mutant, would be a welcome addition for combinational treatment options for anti-cancer therapy. These molecules form a promising base for further development to join the fight against cancer. The development of catalytic Tdp1 inhibitors might also help patients with other diseases such as SCAN1, which appears to be a common founder mutation in the Arab population and even Lupus nephritis^[29,33,52]^. Hence, the “SCAN1” Tdp1H493R mutant enzyme performs the first step [Figure 1] with similar kinetics as the wild type Tdp1^[26]^. However, the rate of the second step is dramatically reduced, resulting in a prolonged life-time of the toxic enzyme-DNA covalent reaction intermediate. Thus, catalytic inhibition of this SCAN1 Tdp1 mutant would prevent the formation of the toxic Tdp1^SCAN1^-DNA intermediate and the subsequent induction of cerebellar atrophy, which could stabilize disease progression and symptoms.

## Declarations

### Copyright

© The Author(s) 2019.
